# Supplementary material for: Beware (Surprisingly Common) Left-Right Flips in Your MRI Data: An Efficient and Robust Method to Check MRI Dataset Consistency Using AFNI
Source: Front Neuroinform. 2020 May 25;14:18. doi: 10.3389/fninf.2020.00018 (PMC7263312; doi:10.3389/fninf.2020.00018)
Supplement: Supplementary file 1 [file Data_Sheet_1.pdf]

APPENDIX A Notes on AFNI commands

Visualization:

Visualization methods for comparing two datasets in the AFNI GUI ; note that some features are more useful for one case or the other, and several include user interactions in the GUI, as described below). In the first column, one can reduce the opacity of the overlay (olay) volume and investigate where tissue classes appear to overlap (the olay colorbar and range can also be adjusted, as convenient). The second column shows how the underlay (ulay) can be toggled with an "edgified" version of itself using a keypress; this is particularly useful, as often one judges alignment by a comparison of structural boundaries. The third and fourth columns show how one can view both volumes side-by-side, sliding the control bar that appears at the top to control where the boundary (either vertical or horizontal, respectively) appears. In the fifth column the two volumes are blended, with the control bar controlling the relative fraction between 0-100% for the olay. Finally, the sixth column shows how the volumes can be displayed in alternating "checkerboard" squares (this is mostly useful for similar volumes, such as in the lower panel). As each of these modes is turned on/off with a simple key press, one can move among them easily. Methods for generating images automatically as part of an analysis pipeline are noted below in Table S1.

The images in Fig. S1 can be displayed automatically in the AFNI GUI by creating a "driver" script; this can facilitate the comparison by automating the GUI behavior (loading specific files, jumping to locations, and performing key presses). Additionally, the images in the first two columns can be created and saved without even opening the GUI (e.g., remotely or running on a "headless" system, to be reviewed at any point), through the use of AFNI scripts and commands. In particular, the *@chauffeur_afni* command creates montages across the FOV and can facilitate making systematic sets of views as part of any processing script (for example, it is the tool that creates the images in *afni_proc.py's* HTML QC, shown in part in Fig. S1).

Processing Pipeline Notes:

The *afni_proc.py* command used to analyze the public datasets in this study is provided in Table S1. Some brief comments on the command:

1) The 'regress' block is only included to generate the QC, treating the data as resting state. No useful time series analysis is carried out in this particular script.
2) The 'volreg' block is mainly included for the 'MIN_OUTLIER' functionality: to find and use the EPI volume that has the smallest fraction of outliers for alignment, since it is likely to not be corrupted by subject motion.
3) The '-giant_move' option is included because several datasets had poor initial EPI-anatomical alignment (typically, large relative translations, as well as rotations); this option implements a center of mass alignment to start, and enlarges the parameter search space (3 shifts, 3 angles, 3 scalings, 3 shearings).
4) The lpc+ZZ cost function leads to slightly slower processing than “pure” lpc, but tends to be the most robust for EPI-anatomical alignment.
5) The EPI datasets in the DS000114 group contained two TRs of pre-steady state values, so the value given to the *-tcat_remove_first_trs* option was 2, instead of 0.

Figure S1. Examples of AFNI GUI features for comparing structural features of two volumes (shown here for axial views, but applicable to all slice planes). Cases where volumes have differing contrasts (T1w-EPI) are compared in the top section, and those with similar contrasts (T2w-DWI) are compared in the lower section. In each case the "original" versions of the volumes appear in the first row, and the versions with a relative left-right flip are shown in the bottom row. In the first column the overlay has opacity set to 4; keypresses are used to enable the features in the remaining columns (from left to right): "e", "4", "5", "6" and "#". Several of the features allow user interaction in the GUI, such sliding the "curtain" boundary between images or toggling views.

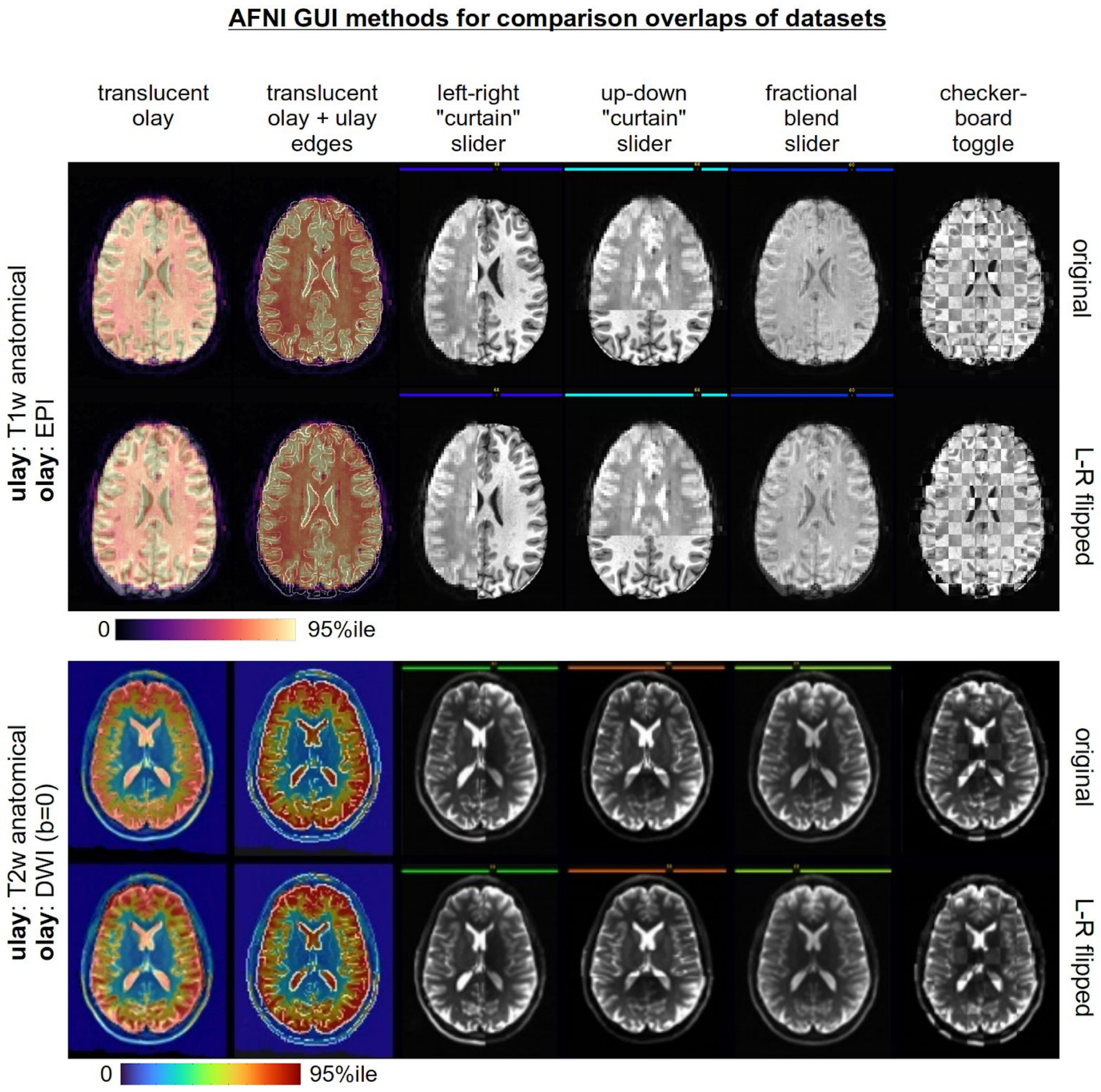

Table S1. The *afni_proc.py* command used to analyze the public data in this study. See comments in the Appendix's text.

```
#!/bin/tcsh

# "mini" afni_proc.py command for performing a left-right flip check;
# to be run for each subject.

set subj    = $1      # subject ID
set topdir  = $2      # "input" directory with subject's datasets
set top_wri = $3      # "output" directory for subject's results

afni_proc.py                                                      \
    -subj_id         ${subj}                                       \
    -script          ${top_wri}/proc.${subj}                       \
    -out_dir         ${top_wri}/${subj}.results                    \
    -dsets           ${topdir}/${subj}/func_*.nii.gz               \
    -copy_anat       ${topdir}/${subj}/anat_*.nii.gz               \
    -anat_has_skull  yes                                           \
    -blocks          align volreg regress                          \
    -tcat_remove_first_trs 0                                       \
    -align_opts_aea        -check_flip -giant_move -cost lpc+ZZ \
    -volreg_align_to       MIN_OUTLIER                             \
    -volreg_align_e2a                                              \
    -regress_run_clustsim  no                                      \
    -html_review_style     pythonic                                \
    -execute
```
